# Understanding vaccine hesitancy with PCV13 in children: Results of a survey in Shanghai, China

**DOI:** 10.1371/journal.pone.0284810

**Published:** 2023-04-27

**Authors:** Yi-hong Ni, Zhen-hui Xu, Jing Wang

**Affiliations:** Department of Immunization Program, Huangpu District Center for Disease Control and Prevention, Shanghai, China; Carol Davila University of Medicine and Pharmacy, ROMANIA

## Abstract

A questionnaire survey for parents of children under 5 years of age was conducted to analyze vaccine hesitancy with the 13-valent pneumococcal conjugate vaccine (PCV13) in Shanghai, China. A total of 892 valid questionnaires were collected. Descriptive statistical methods, Chi-square test and effect size of Cohen were used. Among participants, 421 (48.8%) had children who had been vaccinated with PCV13 before the survey while 227 (26.73%) planned vaccination with PCV13 in the future. The main reasons for not receiving vaccination were the fear of adverse reactions (79, 26.7%), beyond vaccination age (69, 23.3%), and no need to vaccinate (44, 14.9%). Reducing vaccine hesitancy and increasing vaccination willingness can be achieved through health interventions, lower vaccine prices, and the adjustment of vaccination strategies.

## Introduction

Pneumonia is an acute respiratory infection that is most often triggered by viruses or bacteria and can cause mild to life-threatening diseases in individuals of all ages. Moreover, pneumonia is the leading global cause of child death from infectious diseases. In 2017, pneumonia caused more than 808,000 deaths in children under 5 years of age, accounting for 15% of all deaths in this group [1]. China has the second-highest number of pneumonia cases in children under 5 years of age, representing 12% of all cases in the world [2]. Pneumococcal bacteria (Streptococcus pneumoniae, *Spn*) can cause a variety of clinical infections including pneumonia, meningitis, and bacteremia and non-invasive diseases including otitis media, sinusitis and bronchitis—collectively referred to as pneumococcal disease (PD) [3]. Invasive pneumococcal disease incidence in children is being reduced further through universal administration and distribution using broader serotype coverage rather than non-vaccine serotypes, as well as increased vaccination rates [4]. In the World Health Organization (WHO) classification of vaccine-preventable diseases, pneumonia is designated a “very high priority” disease requiring prevention with vaccination. Based on *Spn* capsular polysaccharides, the pneumococcal polysaccharide vaccine and the pneumococcal conjugate vaccine (PCV) cover the most common serotypes that cause PD. By the end of 2020, 151 Member States have introduced PCV and the global coverage of third-dose of PCV is estimated at 49% [5]. The two 13-valent PCVs (PCV13s) in China are produced by Pfizer (PCV 13-Pfizer) and Walwax (PCV13-WX); both are non-immunization program vaccines and are taken voluntarily at the user’s own expense. In China, the per-dose prices of PCV13-WX and PCV13-Pfizer are $88.90 (RMB 598) and $103.80 (RMB 698), respectively [6]. Vaccine hesitancy refers to the reluctance or refusal to be vaccinated despite the availability of vaccines. Vaccine hesitancy is the attitudinal interval between the complete acceptance of vaccines and the complete rejection of vaccines and can lead to lower vaccination rates, thus affecting population immunity [7]. In 2019, the WHO included vaccine hesitancy as one of the 10 threats to global public health [8]. A growing number of individuals are delaying or refusing available and recommended vaccines for themselves or their children [9], potentially reversing the progress made in managing vaccine-preventable diseases. A lack of research on the prevailing vaccine hesitancy with PCV13 in China is apparent. Vaccine hesitancy is complex, with numerous determinants that vary with vaccine type, setting and period in time. Any single strategy is therefore unlikely to be effective in addressing all the determinants of vaccine hesitancy [10]. In this study, we attempted to analyze the prevailing situation of vaccine hesitancy in China. Specifically, we examined the factors that influence vaccine hesitancy with PCV13 in Shanghai to help guide a more targeted vaccination policy that can increase the vaccination coverage of PCV13 and reduce the incidence of pneumonia in children.

## Methods

### Study population

The survey was conducted from September to December 2021 in 10 community vaccination clinics in Huangpu District, Shanghai, China. The survey was physically done with the parents/other family member while they were at the clinic to vaccinate their child with the national schedule vaccines. We used convenient sampling methods. The respondents were asked to voluntarily participate, became a part of the sample and provided relevant information to complete the online questionnaire. Survey respondents were residents who have visited the vaccination clinic during September to December 2021. The respondents were those who visited the vaccination clinic during the survey period. Respondents were required to meet the following criteria. First, respondents were aged 18–65. Usually, parents or grandparents bring their children to the clinic for vaccination. This survey adopts the online questionnaire, older respondents may have some difficulties in answering the questionnaire, so the age limit of respondents is between 18 and 65 years old, which ensured that the majority of the people who came to the vaccination clinic were covered in age, and also ensured the completion of the online questionnaire. Second, respondents had children under the age of five. Since the target group of PCV13 in China is children under 5 years old, the questionnaire needs to investigate the parents of this group. We included only respondents with children under 5 in the study. If the parents had more than one child, the responses to the questionnaire only for the child with whom they were present at the time.

### Questionnaires

There was an introductory text which gave information about the study, the aims of the questionnaire. The anonymous questionnaire for the adult population included questions in the following sections:

♦ Demography (age, gender)
♦ Children’s health and vaccination conditions
♦ Attitudes towards vaccination
♦ Concerns around vaccination

### Data collection

The survey was administered by the staff of the vaccination clinics using online questionnaire app to collect information. Data were anonymized throughout the research. s. In this survey, the respondents scanned the QR code and entered the questionnaire interface, and then completed the questionnaire by themselves. Vaccination history was obtained from the information system or from the children’s vaccination record book. The questionnaire primarily collected general demographic information, children’s PCV13 vaccination history, possible reasons affecting the decision to vaccinate, including individual/group impact, vaccine or immunization program impact, and environmental factors. Vaccine hesitancy was defined as “did not vaccinate with PCV13” or “refused to get vaccinated within the next year” in the case of insufficient vaccine supply. The price was converted to US dollars at the Renminbi–US dollar (RMB/USD) exchange rate of 6.7246, which was based on the central parity of the RMB exchange rate published by the China Foreign Exchange Trade Center in the inter-bank foreign exchange market on July 6, 2022.

### Statistical methods

Descriptive statistics were produced as numbers and percentages. Chi-square test and effect size of Cohen were used for interpretation of findings. The prevailing situation and the causes of vaccine hesitancy were mainly evaluated by examining the three aspects—situation, individual/group influence, and vaccine or immunization program influence-of the WHO’s Vaccine Hesitancy Determinants Matrix [7]. All statistical analyses were conducted using SPSS version 18.0 (IBM, Armonk, NY, USA) and P values < 0.05 were considered statistically significant.

### Ethics approval and consent to participate

This study was approved by the Ethics Review Board of Huangpu District Center for Disease Control and Prevention (No. 2021HPLL08). The first page of the questionnaire included the consent form that explained the research project overview and participant’s confidentiality, making sure that their personal information would remain confidential and they hold the right to withdraw from the study whenever they wish to. Informed consent from participants was obtained before scanning the QR code to complete the questionnaire. Anonymity was guaranteed to participants. All methods were performed in accordance with the relevant guidelines and regulations.

## Results

After 4 months of collection, we received a total of 1,051 valid questionnaires. Among respondents, 293 were male and 758 were female. The 31–40 age group was the largest with 507 (48.2%) respondents, and the most prevalent family role among respondents was “parent” (82.0%). Children’s parents (95.9%) overwhelmingly made vaccination-related decisions (Table 1).

**Table 1:** Demographic characteristics of subjects.

Characteristic (N = 1051)		Number	Percentage (%)
Gender			
	Male	293	27.9
	Female	758	72.1
Age			
	< 25	90	8.6
	26~30	247	23.5
	31~40	507	48.2
	41~50	101	9.6
	51~60	53	5.0
	> 60	53	5.0
Family relationship			
	Parents	862	82.0
	Grand parents	94	8.9
	Others	95	9.0
Decision maker			
	Parents	1008	95.9
	Grand parents	27	2.6
	Others	16	1.5

We believe that only parent or legal guardian responses should have been included in the analysis, and not other family members because a grandparent may not agree to vaccination, but the parent may agree, or another family member may not know the exact reasons why parents are hesitant to vaccinate. So, the analysis of vaccine willingness and hesitancy was done including only the parents’ responses (862 respondents).

### PCV13 vaccination willingness

Before the survey, 421 (48.8%) respondents claimed their children had been vaccinated with PCV13. Among those with a history of PCV13 vaccination, 321 (76.2%) chose Pfizer-PCV13 and 100 (24.4%) chose PCV13-WX.227 (26.3%) respondents planned to have their children vaccinated with PCV13 in the future.

**Fig 1:**
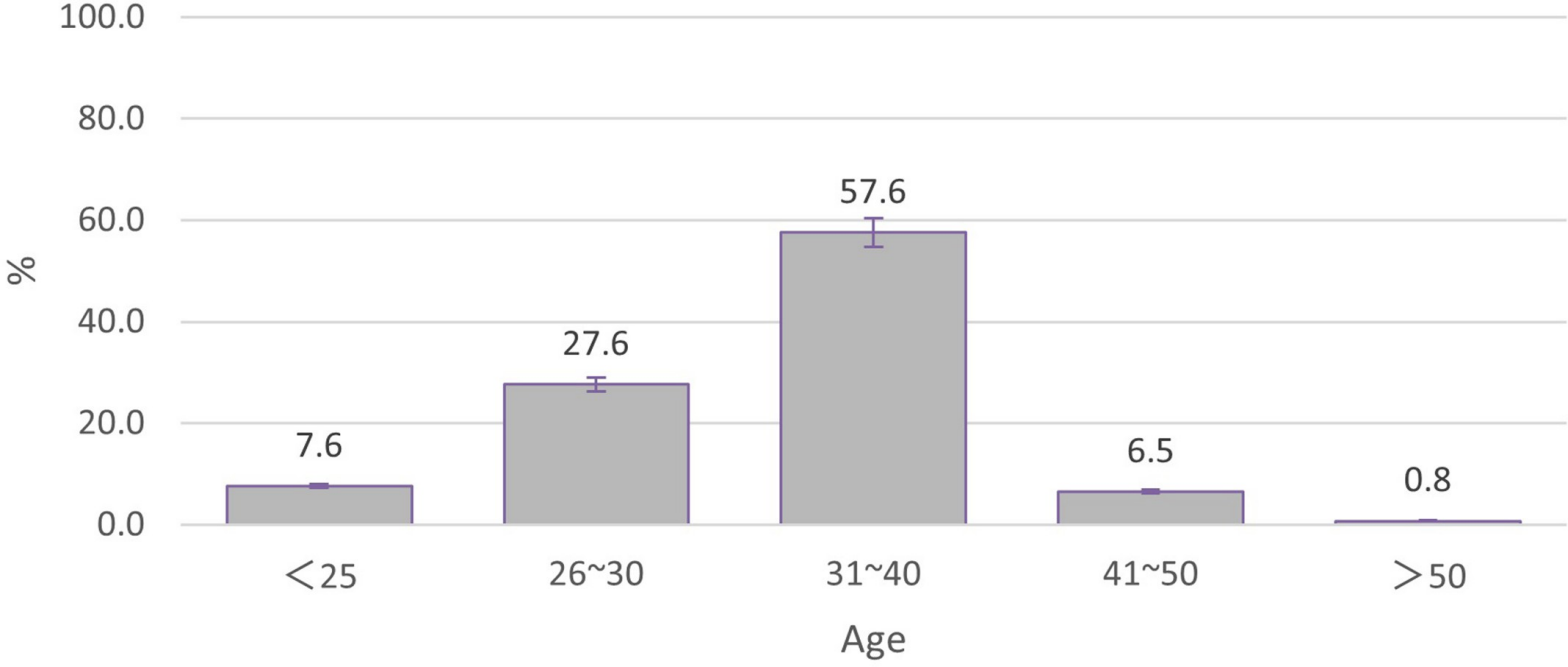
Vaccination history or vaccination willingness in different age groups.

Vaccination history or a willingness to vaccinate PCV13was statistically significant different between the age groups (χ2 = 21.4, p < 0.05; Fig 1). The willingness to vaccinate was analyzed by dividing the parents age into two groups: ≥40 years old and under 40 years old. The effect size of Cohen’s *d* = 0.46, the group of parents less than 40 years old were more willing to vaccinate PCV13. 276 (32.0%) respondents claimed that their children had experienced respiratory diseases in the past year, but the vaccination history or willingness was not related to the history of respiratory diseases. (χ2 = 0.06, p > 0.05, effect size of Cohen’s *d* = 0.02).

### Vaccination hesitancy

The main reasons for choosing Pfizer’s PCV13 were that the vaccine was effective (330, 43.5%) and was recommended by relatives and friends (152, 20.1%) or by a physician (112, 14.8%). The main reasons for choosing Walwax’s PCV13 were the vaccine’s effectiveness (96, 26.5%), doctor recommendation (68, 18.8%), and recommendation by relatives and friends (53, 14.6%). The main reasons for a lack of vaccination were that the fear of adverse events after vaccination (79, 26.7%), children were beyond the age of vaccination (69, 23.3%), and that vaccination was not needed because children were already healthy (44, 14.9%).

**Fig 2:**
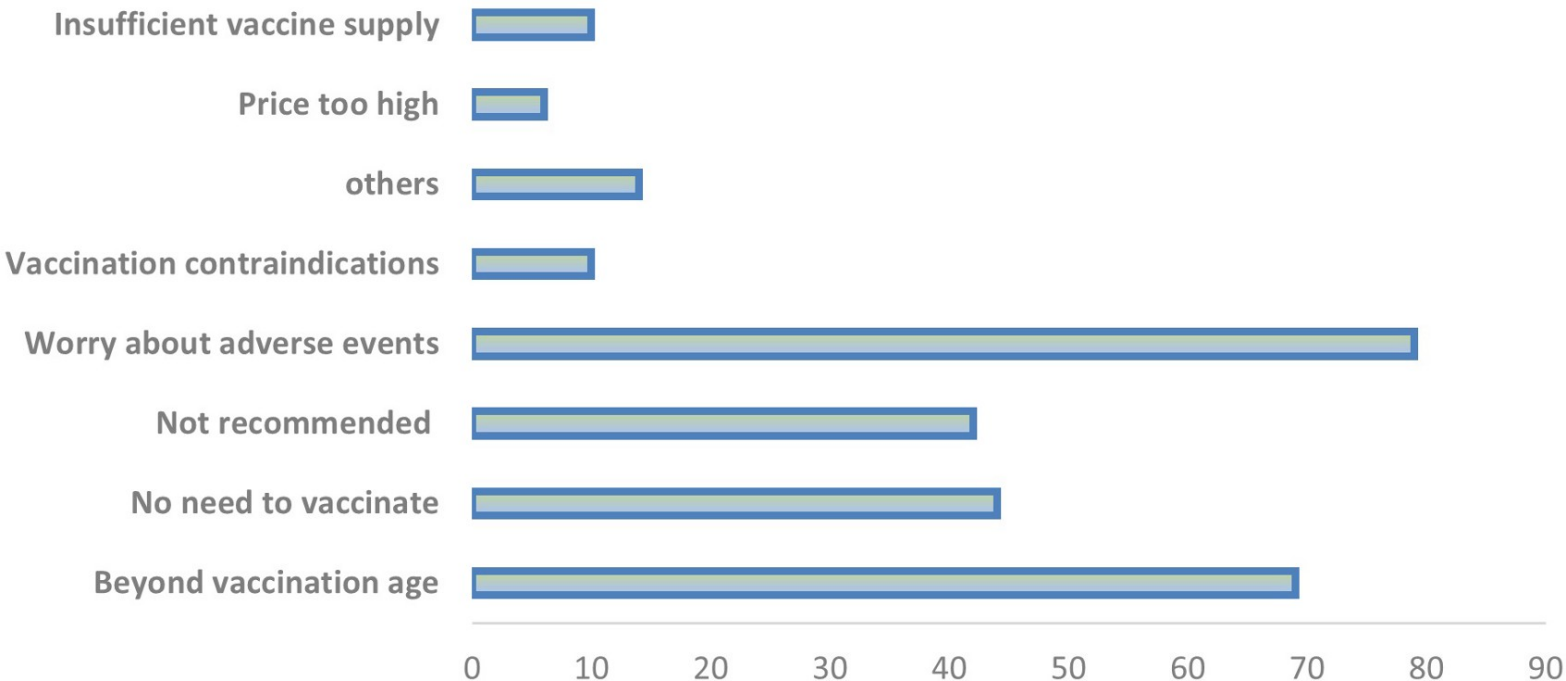
PCV13 vaccine hesitancy in Shanghai, China.

As shown in Fig 2, vaccine hesitancy was classified according to three aspects: 1) individual/group influences, which included “beyond the age range for vaccination” (23.3%), “healthy child does not need vaccination” (14.9%), and “doctor did not recommend vaccination” (14.2%), 2) situational effects including fear of adverse events after vaccination (26.7%), contraindications (3.4%), and other (4.7%), and 3) the impact of the vaccine or immunization program, including factors such as high vaccine cost (2.0%) and insufficient vaccine supply (3.4%).

### Vaccine price

The expected average price for PCV13 per dose was $45.4, and the highest expected price was $104.8, the lowest expected price was $14.9. The expected average price for respondents who had received the vaccination or were willing to vaccinate was $49.6, higher than the $32.6, per dose for those who did not receive the vaccination, the effect size of Cohen’s *d* = 0.65. The expected price for respondents less than 40 years of age was $46.0, higher than the $39.6 for those ≥ 40 years of age, and the effect size of Cohen’s *d* = 0.23.

## Discussion

Receiving vaccination is the result of a multifaceted decision-making process that is likely affected by many factors. In this study, we observed that the 31–40 year age group had the highest proportion of children with a vaccination history or a willingness to vaccinate, and vaccine effectiveness was cited as the main reason for vaccination. This underlines the higher awareness and acceptance of vaccines among the younger population. However, the specific perception of vaccine effectiveness in the young and older non-hesitant sub-groups was not identified. In addition, consistent with the results of global studies on vaccine hesitancy, recommendations from relatives, friends, and doctors were important determinants for choosing vaccination with PCV13 [11]. The fear of the adverse effects of vaccination remained an important determinant of vaccine hesitancy. However, according to multiple studies after the market availability of the vaccine, the incidence of PCV13-related adverse events was not higher than that of other vaccines [12, 13]. Therefore, more effective publicity and targeted communication are needed to address hesitancy and increase vaccination coverage. Vaccine hesitancy can be reduced or eliminated through health transmission interventions and strategies [14].

Although vaccines are recognized as the most effective measure for controlling and preventing disease, an increasing number of individuals believe that vaccination is unsafe and unnecessary. Vaccine hesitancy is weakening individual and community protection against vaccine-preventable diseases [15]. Notably, individuals can now obtain information in multiple ways and the speed of information updates is accelerating. However, the credibility of information cannot be guaranteed. Therefore, enabling access to the latest and most accurate information regarding vaccines through health education and promotional interventions adapted to the modern context is particularly crucial. And perception studies on vaccine hesitancy and behavior have been performed in a timely manner via Social Media tools, reaching rather large cohorts of responding people [16]. Public health messaging should capitalize on social media to provide accessible, transparent, and age-appropriate information. Meanwhile hesitant people are more likely to approach Social media networks than classic communication channels [17]. Face-to-face communication or advocacy may also improve or at least slightly enhance children’s vaccination status and parents’ awareness of and willingness to secure vaccination for their children [18]. Therefore, professionally training medical personnel will effectively improve the communication skills required by health care personnel to clearly and comprehensively answer questions regarding vaccination [19].

Although vaccine price was not a determinant of vaccine hesitancy in this study, the expected price of PCV13 was considerably lower than the price of the two PCV13s currently available in China. Ning Guijun’s cost-effective analysis of PCV13 [20] concluded that the inclusion of PCV13 in the National Immunization Program would be cost-effective at a price below $13.40 (RMB 90) per dose. As such, the price of PCV13 is substantially higher than the cost-effective price, potentially leading to some level of vaccine hesitancy. On the other hand, the daily hospitalization cost for children under 5 years old admitted with pneumonia was $150, and the daily hospitalization cost for severe pneumonia was $241 in Shanghai, China [21]. Furthermore, children vaccinated with at least one dose of pneumonia vaccine had lower hospitalization costs than unvaccinated children [22]. The production of the Walwax PCV13 in China and the introduction of other PCV vaccines in the future may lower vaccine prices, thus reducing the impact of price factors on vaccine hesitancy and making more children eligible for PCV vaccination and protection.

Being outside the vaccination age range was another important determinant of vaccine hesitancy. This is mainly because the two PCV13s currently on the market in China cover a relatively narrow age range and, notably, the initial dose of the Pfizer PCV13 is indicated exclusively in children 6 weeks to 15 months old. In foreign countries, PCV13 use has been extended to adults and older adults [23]. In contrast to the 3 + 1 vaccination scheme adopted in China, Germany changed its PCV immunization scheme from 3 + 1 to 2 + 1 in 2015 given the improvement in national vaccination coverage and the vaccine’s immune effect and cost. The reduction in primary doses has increased vaccination coverage to some extent [24]. Therefore, to improve the demand for vaccines, vaccination strategies in China must consider the needs of residents, vaccine safety, cost, vaccination schemes, and, importantly, the indicated age range for vaccination.

Vaccine hesitancy is complex, with several determinants that vary by vaccine type, external environment, and period in time—underlining that any single strategy is unlikely to effectively address all the factors of vaccine hesitancy [10]. Determinants such as education and socioeconomic status are not unidirectional. For example, a higher education level may increase or decrease vaccine acceptance [25]. A study by Srikanth Umakanthan indicates that the individual’s decision to receive vaccination involves cultural, spiritual, political, and socioeconomic factors [26]. Inaccurate communication can fuel vaccine hesitancy and a major vector of vaccine misinformation is represented by Social Media networks. Debunking in a pro-active approach misinformation cad increase vaccine uptake [27]. Recent data are refuting religiosity as a major driver of hesitancy in rather low socio-economical status communities [28] as opposed to increase of hesitancy in more financial stable but religious groups [29]. Gender-based and generation-based public health policies and communication are recommended because all previously listed factors are generating perception-bias related also to age and gender [30]. To avert misconceptions regarding vaccination in influencing hesitancy, health authorities should openly approach these false claims with both cultural and religious awareness in mind [31]. Tailoring approach is needed in highly polarized communities that are exposed to divergent messaging from authorities [32] or in subgroups with distinctive behavioral or perception patterns [33].

Some regions in China have included the Walwax PCV13 in their local immunization programs and implemented a free PCV13 vaccination strategy. A study showed that the degree of vaccine hesitancy of parents in Shanghai was lower than that of parents in other regions of China [34]. We therefore hope that PCV is included in Shanghai’s local National Immunization Program shortly and that vaccination strategies are rapidly implemented to benefit more children.

## Limitations

This study had several limitations. First, survey respondents were parents of children who were vaccinated in public medical institutions; private clinics were not included in the study. The vaccine hesitancy of respondents in private clinics may be different from that in the general population. Second, this study was conducted in Shanghai, which may not represent vaccine hesitancy in other parts of China. Third, China has been conducting large-scale population-based vaccination with COVID-19 vaccines since 2021. Whether the COVID-19 vaccination policy has affected public attitudes and vaccine hesitancy requires further study. Finally, the impact of vaccine resistance, which is not widely discussed in Chinese society, must be considered.

## Conclusion

Because current age limits for PCV13 in local regulations are generating a significant percentage of hesitancy, schedules could be adjusted to apply to more age groups. In contrast, vaccine hesitancy caused by concerns about vaccine safety and efficacy must be addressed with more publicity and education by means of Mass Media and Social Media, in order to reflect behavioral changes of population regarding medical literacy induced by pandemic events. Suitable strategies and promotion by the government and the healthcare sector are needed to raise public awareness of vaccines.

## Supporting information

S1 File (XLSX)
